# Supplementary material for: From sequence to enzyme mechanism using multi-label machine learning
Source: BMC Bioinformatics. 2014 May 19;15:150. doi: 10.1186/1471-2105-15-150 (PMC4229970; doi:10.1186/1471-2105-15-150)
Supplement: Additional file 2 — Java code of ml2db. Additional file ml2db_code.tar.gz contains the Java source code to run the multi-label machine learning experiments and save the results to database. The code’s Javadoc is included. [file 1471-2105-15-150-S2.zip › additional file 2/ml2db/ml2db/doc/mulan/evaluation/loss/OneMinusAveragePrecision.html]

OneMinusAveragePrecision


JavaScript is disabled on your browser.


- Overview
- Package
- Class
- Use
- Tree
- Deprecated
- Index
- Help

- Prev Class
- Next Class

- Frames
- No Frames

- All Classes

- Summary:
- Nested |
- Field |
- Constr |
- Method

- Detail:
- Field |
- Constr |
- Method


mulan.evaluation.loss

## Class OneMinusAveragePrecision

- java.lang.Object
- - mulan.evaluation.loss.RankingLossFunctionBase
  - - mulan.evaluation.loss.OneMinusAveragePrecision

- All Implemented Interfaces:
  :   java.io.Serializable, MultiLabelLossFunction, RankingLossFunction

  ---

    

  ```
  public class OneMinusAveragePrecision
  extends RankingLossFunctionBase
  ```

  Implementation of the average precision as loss function.

  Version:
  :   2010.12.14

  Author:
  :   Grigorios Tsoumakas

  See Also:
  :   Serialized Form

- - ### Constructor Summary

    Constructors

    Constructor and Description

    `OneMinusAveragePrecision()`
  - ### Method Summary

    Methods

    | Modifier and Type | Method and Description |
    | --- | --- |
    | `double` | `computeLoss(int[] ranking, boolean[] trueLabels)` Computes the ranking loss function |
    | `java.lang.String` | `getName()` Returns the name of the loss function |

    - ### Methods inherited from class mulan.evaluation.loss.RankingLossFunctionBase

      `computeLoss`
    - ### Methods inherited from class java.lang.Object

      `equals, getClass, hashCode, notify, notifyAll, toString, wait, wait, wait`

- - ### Constructor Detail


    - #### OneMinusAveragePrecision

      ```
      public OneMinusAveragePrecision()
      ```
  - ### Method Detail


    - #### getName

      ```
      public java.lang.String getName()
      ```

      **Description copied from interface: `MultiLabelLossFunction`**

      Returns the name of the loss function

      Returns:
      :   the name of the loss function


    - #### computeLoss

      ```
      public double computeLoss(int[] ranking,
                       boolean[] trueLabels)
      ```

      **Description copied from interface: `RankingLossFunction`**

      Computes the ranking loss function

      **Specified by:**
      :   `computeLoss` in interface `RankingLossFunction`

      **Specified by:**
      :   `computeLoss` in class `RankingLossFunctionBase`

      Parameters:
      :   `ranking` - the ranking of the learner for an example
      :   `trueLabels` - the ground truth of the example

      Returns:
      :   the value of the loss function


- Overview
- Package
- Class
- Use
- Tree
- Deprecated
- Index
- Help

- Prev Class
- Next Class

- Frames
- No Frames

- All Classes

- Summary:
- Nested |
- Field |
- Constr |
- Method

- Detail:
- Field |
- Constr |
- Method
